# Teamwork: A Systematic Review of Implications From Psychosocial Constructs for Research and Practice in the Performance of Ultimate Frisbee Games

**DOI:** 10.3389/fpsyg.2021.712904

**Published:** 2021-08-27

**Authors:** José Pedro Amoroso, Ricardo Rebelo-Gonçalves, Raul Antunes, Jay Coakley, Pedro Teques, João Valente-dos-Santos, Guilherme Eustáquio Furtado

**Affiliations:** ^1^Department of Human Kinetics, Polytechnic Institute of Leiria, Leiria, Portugal; ^2^CIEQV, Life Quality Research Centre, Polytechnic Institute of Leiria, Leiria, Portugal; ^3^CIDAF, The Research Unit for Sport and Physical Activity, Faculty of Sport Sciences and Physical Education, Coimbra, Portugal; ^4^ciTechCare, Center for Innovative Care and Health Technology, Polytechnic of Leiria, Leiria, Portugal; ^5^Department of Sociology, University of Colorado, Colorado Springs, CO, United States; ^6^School of Social Sciences, Education and Sport, Polytechnic Institute of Maia, Maia, Portugal; ^7^N2i, Research Nucleus, Polytechnic Institute of Maia, Maia, Portugal; ^8^CIPER, Interdisciplinary Center for the Study of Human Performance, Lisbon, Portugal; ^9^CIDEFES, Centro de Investigação em Desporto, Educação Física e Exercício e Saúde, Universidade Lusófona, Lisboa, Portugal; ^10^UICISA:E, Health Sciences Research Unit: Nursing, Nursing School of Coimbra, Coimbra, Portugal

**Keywords:** teamwork, communication, task cohesion, flying disc, sport psychology

## Abstract

**Introduction:** Ultimate Frisbee (UF) is a non-contact, challenging, and self-promoted team sport. Some factors such as the game environment and rules seem to influence athletes' behavior. Goals: Provide a robust systematic review (SR) of the psychological domains associated with UF.

**Methods:** A SR according to Cochrane guidelines was completed. A reproducible search strategy was conducted by two independent reviewers in thirteen online databases: the Cochrane Central Register of Controlled Trials, Web of Science, SCOPUS, B-On, SportDiscus, Scielo; APA PsycINFO, Psychology and Behavioral Sciences; Academic Search Complete; Medline (PubMed); ERIC; Google Scholar; Open Acess Thesis and Dissertations. The search occurred from 1st to 30th June 2020, and there were no limitations regarding the year of publication. Original papers that contained relevant data regarding psychological domains in the context of UF in English, Portuguese and Spanish were selected. The combination of the main terms “ultimate frisbee” and “sport psychology” was used in all databases. A total of 464 studies were identified and selected in the last phase of selection. After the Screening (*n* = 301) and Eligibility (*n* = 71) phases, a total of 30 potential papers were selected and classified. Finally, only four papers were qualified to be included in the final version of SR.

**Results:** The psychological dimensions revealed in the present study were: leadership; basic psychological needs; behaviors; task cohesion and performance; intrateam communication; performance-avoidance goals; friendship goals; sportsmanship associated with goal-directed self-talk and self-regulated learning.

**Discussion:** To our knowledge, this is the first SR about UF. In reviewing all the findings in the studies, there is evidence that UF can promote teamwork, task cohesion, leadership, and increase friendship-approach goals.

**Conclusion:** The results revealed that group goals and promoting teamwork significantly predicted social cohesion and that teamwork and task cohesion was mediated by communication. UF is characterized by communication between all players, whether they are from the same team or the opposing team. In summary, the current study revealed real-time information about the game and its rules. This is important because UF is one of the few team sports worldwide that are self-referred by participants.

**Systematic Review Registration:**https://www.crd.york.ac.uk/prospero/display_record.php?RecordID=169294, identifier: CRD42020169294.

## Introduction

The first complete description of Ultimate Frisbee (UF), including general and specific rules, equipment, time, scoring, game variations, and other characteristics were presented by Clark et al. (1981). From then on, UF was highlighted as an attractive alternative to traditional team sports in physical education classes, and a pedagogical sequence (called Ultimate Curriculum) for introducing this sport modality in the context of United States of America schools (Caporali, 1988) was even suggested. Currently, UF is one of the fastest-growing team sports (Piepiora et al., 2020) and the attempt to promote this sport has led some experts to highlight its qualities related to the development of cognitive, psychomotor, and affective skills at different levels, in addition to the cardiovascular fitness (Clark et al., 1981; Caporali, 1988).

Traditionally known as “Ultimate” among participants, UF is a fast-paced, non-contact, mixed team sport played with a flying disc or frisbee (Griggs, 2011), assembling features of several invasion games, such as American football and netball, into a simple and demanding game (Spencer-Cavaliere et al., 2017). According to the annual census completed in 2019 by the World Flying Disc Federation (WFDF), the largest national member federation is the United States of América (USA), followed by Canada, Australia, Germany, Great Britain, and Japan. There are 86 active member associations, and 176,134 active players, 38% of which are women (World Flying Disc Federation, 2019; Koeble and Seiberl, 2020).

The rapid growth of registered UF practitioners in recent years has attracted interest among researchers in the sport sciences and other disciplines. The published literature on UF has generally focused on: (i) physical, cardiovascular and metabolic demands in healthy adults and athletes (Krustrup and Mohr, 2015; Weatherwax et al., 2015; Leicht et al., 2019); (ii) gender differences among school and university players (Neville, 2019; Piepiora et al., 2020); (iii) sociological analysis associated to rules, ethics and competitiveness among the UF practitioners (Griggs, 2009b; Crocket, 2015, 2016); throwing biomechanics, disc trajectory and injury prevention (Akinbola et al., 2015; Koeble and Seiberl, 2020).

UF has many distinguishing features when compared to other team sports. These include self-arbitration, self-regulation, and independent communication. The UF is self-referred even at the world championship level, and players are expected to stand by a moral code of fair play, called *Spirit of the Game* (SOTG) (Crocket, 2015). The SOTG reveals these characteristics, and to some extent, appears to modulate behaviors, actions, and some psychological aspects of the game (Spencer-Cavaliere et al., 2017). For example, it is reported that the SOTG promotes the following (Clark et al., 1981): (i) competitive play combined with mutual respect between all players; (ii) play for pleasure and joy; (iii) rejecting actions such as provoking opponents, intentional aggressions, and “win at all costs” behaviors – all of which comprise the psychological dimension of UF according to some researchers (Griggs, 2009b, 2011).

Research conducted by Méndez-Giménez et al. (2015) found that the SOTG is also associated with other psychological domains, such as sportsmanship, social goals, and friendship goals. Another study evolving 60 players (30 men and 30 women), reported that the personality traits of UF players differed in levels of neuroticism and that women had higher neuroticism than men (Piepiora et al., 2020). Using qualitative research methods (Robbins, 2012), revealed that cooperation in UF is due to the sport being federally recognized, thus promoting the regulation of competitions through norms, reputations, and self-discipline.

Psychological factors and their influence in the environment of UF have led researchers and practitioners to observe and take note of the impact of the SOTG during competitive events (Robbins, 2012) and its impact on the psychological characteristics of athletes in this sport (Knutson and McAndrew, 2016). To our knowledge, there is little empirical evidence related to the psychological domains in UF. In this sense, the objective of this systematic review (SR) is to consider research trends on UF and related psychological domains, involving the game's characteristics, such as self-arbitration, SOTG, and the game environment. Thus, this SR aims to provide psychological insights into the nature of the sport UF. We believe this study will generate further interest in UF within the sports science community, as this sport has been neglected so far (Lam et al., 2021). Furthermore, the identification and analysis of UF characteristics and environment can be used to enhance players/students and team's performance.

## Methods

To guarantee consistency, accuracy, and replicability in this SR, the following steps were adopted: (i) definition of systematic search terms through the description and operationalization of concepts; (ii) a pilot study of the systematic search of articles to verify the search accuracy in each previously selected database; and (iii) registration of the pre-determined SR protocol in the PROSPERO database, under the number CRD42020169294.

### Description of Main Concepts

a) Ultimate frisbee: Ultimate is a team sport where contact between players is not allowed. It is played by two, seven-person teams, and it can be played with gender-mixed teams. The official field measures 64 meters by 37.57 meters, with 22.86 meters end zones. Each game is played for 48 min and is divided into two 24-min halves (Caporali, 1988). Because the game is self-refereed, tolerance requires players to give up a possible dishonest benefit (Crocket, 2015).
b) Sport and exercise psychology is the scientific study of people and their behaviors in sport and exercise contexts and the real application of that understanding (Gill and Williams, 2008). Researchers work to recognize how the psychological factors associated with practical behavior inspire physical performance, and how the influence of participation in these activities could affect the well-being emotional development, and health of a person in that ecosystem (Tanaka and Sekiya, 2010).
c) Sport and exercise psychology dimensions: the field of exercise psychology has tended to grow from sport psychology and sport science to become an increasingly important topic in health research and is now associated with areas such as health psychology (Biddle and Fuchs, 2009; Lindahl et al., 2015). Some of the most studied dimensions include self-perception and personality (i.e., self-confidence, personality traits, leadership behavior), cognition (i.e., team communication), mood states (i.e., stress, anxiety, motivation), leadership, communication, and team cohesion (Weinberg and Gould, 2014).

### Pilot Search

Previous knowledge of studies of the UF and the pilot search led us to a search focused on the psychological dimensions, due to the number of articles produced related to UF. This preliminary stage of the study was carried out to verify which preliminary results would be generated using the previously selected terms in a combined or isolated manner. The search strategy was based on the descriptor terms and keywords, “frisbee,” or “flying disc,” combined with the terms “ultimate Frisbee,” indexed to the medical subject headings (Huang et al., 2011). In the first search, we used only the term “frisbee” and we identified 11,792 results. In the second search, the combination “frisbee” OR “flying disc” was used, and 11,627 results emerged. In the sixth search, we entered the term “ultimate frisbee” and 2,512 papers were identified.

After this process, the research team decided that improving the accuracy of the search in the different databases required that the terms should be previously selected, since the tools to assist advanced meta-search change depending on each database. Finally, the keywords defined in concordance with all the authors were: “ultimate frisbee” AND “sports psychology.” During this phase, possible additional terms that could be accessed in search assistants were also checked; however, no additional terms in the literature on the topic improved the search profile. Table 1 shows the key terms used in the respective databases in this phase, taking into account the number of articles generated from the different entries with the isolated or combined terms.

**Table 1:** Search terms used depending on the different databases and the number of articles generated in the pilot search.

**Data bases**	**“frisbee”**	**“flying disc”**	**“frisbee” OR “flying disc”**	**“frisbee” OR “flying disc” OR “disco voador”**	**“frisbee” OR “flying disc” AND “sports”**	**“ultimate frisbee”**	**“ultimate frisbee” AND “psychology”**	**“ultimate Frisbee” AND “sport psychology”**
PUBMED (Medline)	260	6	262	1,425	31	17	0	0
WEB OF SCIENCE	213	22	212	212	197	53	1	1
SCOPUS	220	25	238	238	70	53	4	4
APA PsycInfo	43	2	45	68	43	15	8	6
B-ON	9,821	235	10,005	10,041	9,821	1,963	720	428
ERIC	30	0	30	31	30	10	2	2
SportDiscus	92	6	96	102	94	48	10	10
Psychology and Behavioral Sciences	11	2	11	13	11	2	2	2
Academic Search Complete	335	11	342	880	338	42	9	9
SCIELO	5	5	5	11	3	0	0	0
Cochrane Central	7	1	8	9	0	0	0	0
Google Scholar	678	284	291	1	1	277	0	0
Open Access Thesis and Dissertations	77	68	82	84	5	32	2	2
Total of records	11,792	1,318	11,627	13,115	10,644	2,512	745	464

### Search Strategy

After the identification of key terms, an exhaustive and systematic search was performed. A comprehensive, reproducible search was conducted in English (published or in press) across thirteen online databases: (i) the Cochrane Central Register of Controlled Trials, (ii) Web of Science, (iii) SCOPUS, (iv) B-On, (v) SportDiscus, (vi) Scielo; (vii) APA PsycINFO, (viii) Psychology and Behavioral Sciences; (ix) Academic Search Complete; (x) Med line (PubMed); (xi) ERIC; (xii) Google Scholar; (xiii) Open Access Thesis and Dissertations. Original articles (exploratory, cross-sectional); interventional (quasi-experimental and Interventions) published between 1960 and 2020 investigating the associations between UF and different psychological dimensions were selected. The research procedures were carried out between the 1st to the 30th of June 2020 by the first author, guided by the last author, who coordinated the SR.

### Selected Manuscripts Criteria

The initial search was conducted by two researchers who used a list of terms and keywords. The subsequent screening procedures were implemented to determine whether the articles from the initial search were significant for the study. The selected articles in the present QSR met the following selection criteria: (i) original research published in peer-reviewed online international journals indexed in all databases previously identified (excluded were letters to the editor, abstracts in conference proceedings, and systematic review articles of any kind); (ii) the articles should contain one or more keywords in the title or abstract to proceed to the screening phase; (iii) reading of the article in full-text and discussion with other experts on the topic. Articles classified as “distrustful,” but already in the eligibility phase; (iv) were considered articles of open or closed access. In the case of closed access articles, direct contact was made with one of the authors to obtain the full version of the manuscript.

### Data Extraction

**Figure 1:**
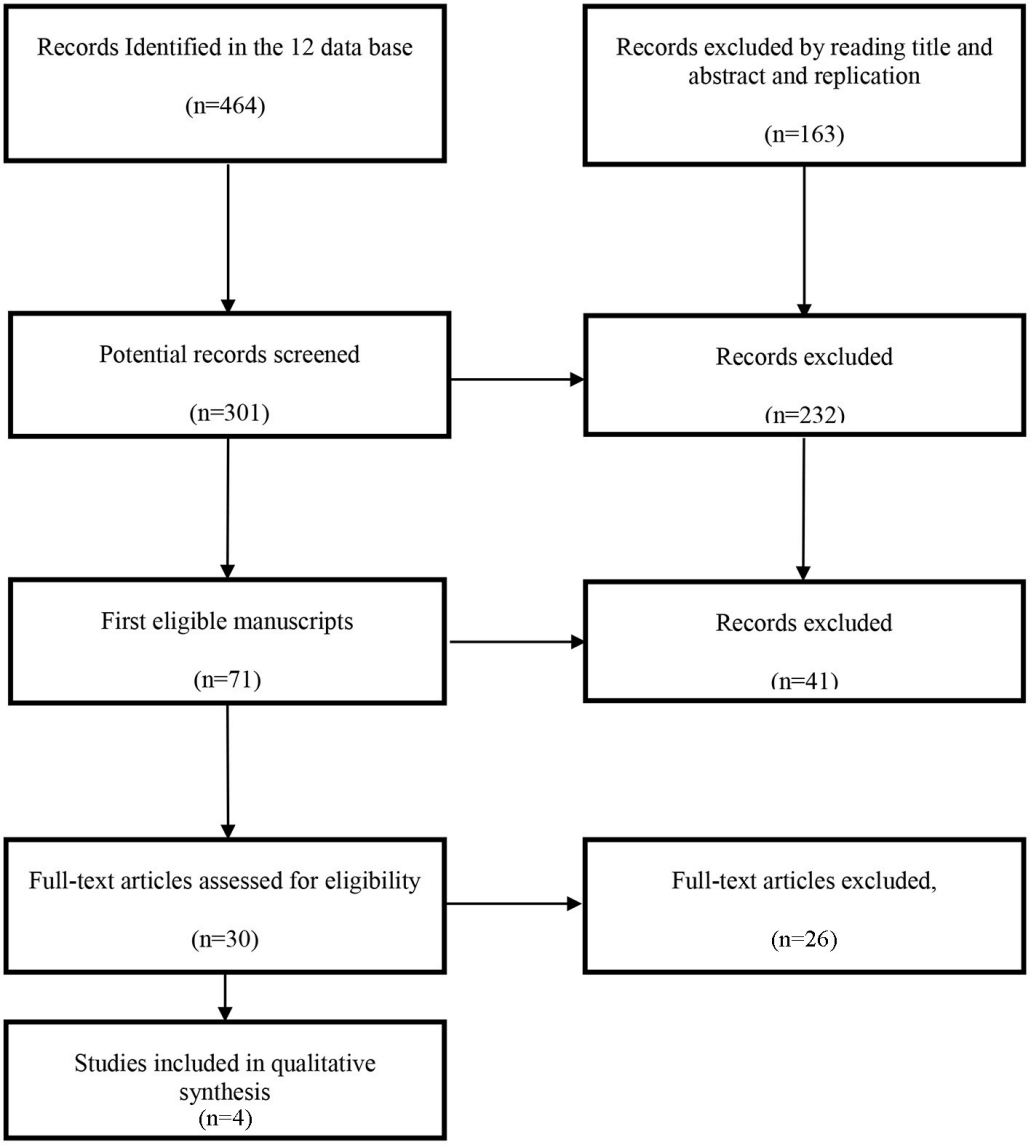
Flowchart of studies included following PRISMA guidelines.

The Selected Reporting Items for Systematic Reviews and Meta-Analyses (PRISMA) Statement for the organization of this study was respected (Liberati et al., 2009; Moher et al., 2015). The guidance of PRISMA describes four specific stages (identification, screening, eligibility, final selection) necessary to implement the search and selection of manuscripts under an SR and feature the flowchart which indicates the respective final selection phases of the studies (Figure 1).

PRISMA guidelines present the PICO acronym (“population,” “intervention,” “comparison, comparison,” “outcomes”), which directs the improvement of the systematic search, operating the extraction. Table 2 identifies the characteristics of the present study, considering the adapted version of acronym PICO guidelines.

**Table 2:** Presentation of the characteristics of the studies included in the review according to of adapted PICOS guidelines.

**Acronym**	**Information**
P	Athletes (age, gender, categories, physical condition, school sport, university sport, sport club)
I	The game (or tournament/championships) of Ultimate Frisbee
C	The Ultimate Frisbee game and/or the subgroups of independent variables
O	Characterized psychological dimensions (i.e., leadership, behaviors, cohesion, performance, goal orientation, sportsmanship)

### Quality of Assessment

In addition, the Strengthening Reporting of Observational Studies in Epidemiology (STROBE) Positioning Statement was used (Von Elm et al., 2008). This method consists of a checklist containing 22 items (100%), which characterizes each study based on the quality assessment that it presents. In this SR, a mutual model of study designs, which is specifically assessed, epidemiological, observational, population-based, cross-sectional, or cohort studies were used (Abeysena, 2011). The purposefulness of this process was not to use traditional cut-off points to be included, or not included some papers in the SR. In its place, the percentage value was used to identify studies in which little quality assessment could affect the quality of SR evidence. Table 3 shows the summary of evidence of all studies included in the SR.

**Table 3:** Quality assessment of selected papers using STROBE - combined check-list.

	**Title and abstract**	**Introduction background/rationale**	**Introduction - objectives**	**Methods: study design**	**Methods: settings**	**Methods: participants**	**Methods: variables**	**Methods: data sources/measurement**	**Methods: bias**	**Methods: study size**	**Methods: quantitative variables**	**Methods: statistical methods**	**Results participants**	**Results: descriptive data**	**Results: outcome data**	**Results: main results**	**Results: other analyses**	**Discussion: key results**	**Discussion: limitations**	**Discussion: interpretation**	**Discussion: generalisability**	**Funding**	**Sum items**	**Total percentage of items**
**Items (paper session)**	**I**	**II**	**III**	**IV**	**V**	**VI**	**VII**	**VIII**	**IX**	**X**	**XI**	**XII**	**XIII**	**XIV**	**XV**	**XVI**	**XVII**	**XVIII**	**XIX**	**XX**	**XXI**	**XXII**	**22**	**100%**
1. Callow et al. (2009)	1	1	1	1	1	1	0	1	0	1	1	1	1	1	1	1	1	1	1	1	1	0	19	86%
2. Smith et al. (2013)	1	1	1	1	1	1	1	1	0	1	1	1	1	1	1	1	1	1	1	1	1	0	20	91%
3. Méndez-Giménez et al. (2015)	1	1	1	1	1	1	0	1	0	1	1	1	1	1	0	1	1	1	1	1	1	0	18	82%
4. Latinjak et al. (2018)	1	1	0	0	1	0	0	1	0	1	0	1	1	1	1	1	0	0	1	1	0	0	11	50%

*Met the criteria yes = 1; no = 0*.

### Data Analysis and Risk of Bias

An SR search method was performed to identify all possible data for this review following Cochrane guidelines, considering all the previous criteria. A single reviewer (first author) checked the list of articles and discarded irrelevant hits based on title and abstracts. Then, two reviewers (penultimate and last authors) selected, independently, those papers that fulfilled inclusion criteria. Subsequently, the risk of bias was assessed for each study using Cochrane criteria. Any disagreement was resolved by discussion with all authors. During the process of constructing the SR (mainly considered the pilot search phase), it was found that the evidence gathered did not allow us to select a central outcome to proceed to an SR with meta-analysis. At the end of the search, the small number of selected articles corroborated this point.

## Results

### Results of Meta-Search

A total of 464 references were identified through the database in the first phase. Out of these, 163 references were excluded after reading the title and abstract, and replication. After applying these initial criteria, a total of 301 articles entered phase two of eligibility. Of these, 232 papers were later excluded for reasons such as “dealt with other similar modalities,” or “approached study dimensions of different nature,” among others. After the full text of articles was assessed, a total of 71 articles remained eligible, 41 of which were excluded, mainly because they used qualitative research methods. In the last phase of Inclusion, all authors decided that only articles that have psychological dimensions would be included in the final SR, considering the previously presented concepts. As a result, 26 studies were excluded at this stage because they presented social or psychosocial approaches that could cause bias in the presentation of results. In total, four studies were included in the final version of SR.

### General Characteristics of Selected Studies

Table 4 shows the general characteristics of all studies included in this SR. Of the four selected studies, two were characterized as intervention studies (Méndez-Giménez et al., 2015; Latinjak et al., 2018), and two used a cross-sectional design (Callow et al., 2009; Smith et al., 2013). A total of 895 UF players of both genders (*n* = 367 female; *n* = 528), from four different countries (Britain, United Kingdom, Spain, and Poland) participated in these four different studies. Different levels of UF players can be observed across the selected studies such as novice players, student players, university players, and team players. We also observed that the participants' age generally varies between 20.77(±2.03) and 24.30(±3.90) years. The experience of regular and deliberate practice of UF with the same captain varies between 1.25 (±1.30) years in the different selected studies. Lastly, we noted that leadership behaviors (Callow et al., 2009), leadership and task cohesion (Smith et al., 2013), motivation and sportsmanship (Méndez-Giménez et al., 2015), goal-directed self-talk and performance (Latinjak et al., 2018), and personality profile (Piepiora et al., 2020) were the sport psychological dimensions investigated in the four selected studies.

**Table 4:** Summary of reviewed studies.

**References**	**Sample**	**Age (M ± SD)**	**Type of study**	**Measures**	**Main findings**
1. Callow et al. (2009)	309 club standard ultimate Frisbee players in the United Kingdom	24.30 ± 3.90	Cross-sectional study	Transformational leadership, cohesion and performance levels of study participants	High evidence for the validity of the Differentiated Transformational Leadership Inventory (DTLI) and high relationship between specific transformational leadership behavior's and both cohesion and level of performance.
2. Smith et al. (2013)	199 university level ultimate Frisbee players (199 participants (male 110, female 89)	20.77 ± 2.03	Cross-sectional study	Transformational leadership, intrateam communication and Team cohesion	The differentiated model of transformational leadership allowed identification of specific leadership behaviors that predict both intrateam communication and task cohesion; Training to develop specific leadership behavior's; leader training to improve intrateam communication, might be an intervention to increase the task cohesion of sports teams.
3. Méndez-Giménez et al. (2015)	295 secondary school students	14.2 ± 1.68	Interventional study (A quasi-experimental design)	Mastery, performance, friendship, autonomy, competence, relatedness, Social conventions, rules and officials and opponent.	The Sport Education (SE) model has been proven more efficient that a Traditional teaching approach to develop the best valanced achievement goals and social goals, to fulfill students' basic psychological needs and to promote fair play;
4. Latinjak et al. (2018)	32 novice Ultimate Frisbee players	22.88 ± 9.71	Interventional study	Instructional self-talk	interactions between instructional self-talk content and performance outcomes; Athletes in self-talk intervention should not only create and use self-talk plans, but also learn to adapt their cue words to forthcoming actions as well as past, successful and unsuccessful, attempts. The results of this study suggested that several relevant psychological constructs can be expressed by athletes via self-talk; Coaches who learn to listen carefully to their athletes' goal-directed self-talk might gain additional insight regarding their personality.

### Specific Characteristics of Selected Studies

In the first cross-sectional exploratory study, involving a sample of 309 (24.3 ± 3.9 years old) UF players (female: *n* = 105; male: *n* = 204), the results indicated that the leadership behaviors of fostering acceptance of group goals and promoting teamwork, high-performance expectations, and individual consideration significantly predicted task cohesion (Callow et al., 2009). In this study, the authors verified that the results offered support for the factorial and discriminant validity of the Differentiated Transformational Leadership Inventory (DTLI) questionnaire. Later, the DTLI questionnaire had its final validation processes completed (Smith et al., 2013).

The second cross-sectional study, including 199 UF university players aimed to analyze the instruments that may mediate the connection between transformational leadership behaviors and follower outcomes in the sporting domain (Smith et al., 2013). The results showed that the relationship between individual consideration and task cohesion was intermediated by communication. In addition, the relationship between fostering acceptance of group goals, teamwork, and task cohesion was mediated by communication. Elevated performance expectations were found to be strongly related to task cohesion and it was not correlated to any of the sub-dimensions of communication. Essentially, the authors concluded that transformational leader behaviors straight are directly related to group outcomes such as cohesion (Smith et al., 2013). In this analysis, two questionnaires were used: the Scale for Effective Communication in Team Sports-British (SECTS-B; Sullivan and Callow, 2005), to assess “intrateam communication”; and the Group Environment Questionnaire (GEQ; Carron et al., 1985) to examine “team Cohesion.”

In the third interventional study, involving a total of 295 secondary school students, aged 12–17 years old, the analysis indicated that a mastery-approach and friendship-avoidance goals constituted the main score, while both performance goals achieved the lowest scores in this specific group of students (Méndez-Giménez et al., 2015). The results also indicated that all interventions increased friendship-approach goals. In conclusion, the sports education model was proven to be more proficient than a traditional teaching approach to improve the most balanced achievement goals and social goals, to fulfill students' basic psychological needs, and to promote fair play. According to this study, authors applied questionnaires in two sessions with 30 min before and after the completion of a 12-week intervention program. The following questionnaires were applied: (i) the Achievement Goals Framework (Elliot and McGregor, 2001); (ii) Friendship goals Questionnaire – Physical Education (Garn and Sun, 2009); (iii) Basic Psychological Needs in Exercise Scale (Méndez-Giménez et al., 2014); (iv) Multidimensional Sportspersonship Orientations Scale (Vallerand et al., 2016).

In the fourth interventional study developed by Latinjak et al. (2018) a total of 32 novice UF players were participants. To verify the results of the intervention, the authors examined goal-directed self-talk in a total of three situations: before a throw, after a successful or unsuccessful throw. During this part of the research, the participants were asked to write as much self-instruction as they considered giving themselves to increase their performance or make progress on the task; (a) before a throw, (b) after unsuccessful throws, and (c) after successful throws. Success and failure were, mostly, determined by the players' subjective performance evaluations, and secondarily, by the effective reception of the frisbee by a team player. According to the same authors, the innovative contribution of this study was the description of differences in the content of instructional self-talk depending on the situation. The results highlight that there is no reason to believe that only UF players use goal-directed self-talk or that no one else uses the categories of self-talk they have used. Also, they suggested that several relevant psychological constructs can be expressed by athletes via self-talk (Theodorakis et al., 2000), motivational self-talk, and instructional produced considerably better performance than a control condition for a strength task. Furthermore, samples of goal-directed self-talk could yield complementary insight to information achieved through the administration of psychometric questionnaires.

### Description of Excluded Studies

The study developed by Piepiora et al. (2020), aimed to determine the personality of the UF players and was the only study excluded in the “included phase.” Despite fulfilling the stipulated classification criteria, this study did not become eligible for inclusion because important elements that helped to understand the main aspects of psychological domains related to UF were omitted according to the combined checklist STROBE statement (Von Elm et al., 2008). No additional information from this study could be obtained, making its characterization difficult. Regarding other studies excluded during the eligibility phase, we identified that they had a scope related to the field of the psychosociological or sociological of sport (Griggs, 2009a; Crocket, 2016; Neville, 2019). Despite having similarities to those defined for inclusion, these excluded studies presented specific characteristics, such as the use of qualitative design as a method of data collection.

## Discussion

This SR aimed to provide psychological insights into the nature of the sport UF. Furthermore, the identification and analysis of UF characteristics and environment can be used to enhance players/students' and team performance. To the authors' knowledge, this is the first systematic review on this topic. The findings revealed a direction for the improvement in the dimensions of leadership, behaviors, task cohesion, group goals, teamwork, social goals, and performance among those who practice UF. This strongly suggests that there are lines of research that seek to highlight UF as a sport with potential in different areas. However, UF is a self-referred sport, and this variable was not included in any of the studies.

UF is a competitive, non-contact, invasion-style team sport played with a flying disc (Thornton, 2004; Griggs, 2011; Crocket, 2015). Cooperation between teammates is a dependent factor for success, which characterizes the internal logic and tactical behavior of players and teamwork theory. With UF we can apply teaching directed to young people so that in a formal or non-formal way they can learn to self-regulate. The implementation of activities intended to improve the level of respect in schools will be valuable in educating sportsmanship behaviors (Koç and Yeniçeri, 2017). This way practitioners have an opportunity to reveal areas of their practice that may be developed, and researchers have new opportunities to consider how they can continue to advance the body of writing.

In reviewing all the findings in the studies, there is evidence that UF can promote teamwork, task cohesion, leadership, and increase friendship-approach goals. As goals affect performance by focusing attention on the task, encouraging persistence, and increasing effort, intensity, and new performance strategies (Vallerand et al., 2016). Teamwork, cooperative play, and good sporting behavior are stressed as important aspects of game playing (Carpenter, 2010).

Future expectations about the SOTG were not mentioned in any of the four selected studies. When Griggs (2011) reviewed how the constitutive rules of UF operated in practice within the ethos of Spirit of the Game, he noted that the high degree of social control within UF was related to self-refereeing and an implied agreement among players to uphold the rules of the sport. Similarly, Thornton (2004) portrayed SOTG as a code of conduct that supported and reinforced self-officiating. As evident in this article, UF and SOTG promote goal setting those structures and organizes an approach to participation that enables youngsters in daily training to define sports competitions with focus and good direction.

Some of them with a qualitative nature that sought to study psychosocial aspects of UF. We are not aware of any other studies, have looked for other dimensions as team leadership would demand that the opposing team leadership rectify the situation either by controlling their teammate or by removing their teammate from play (Robbins, 2012). This is possible because UF is a self-regulated team sport even at the level of International and World Games (Griggs, 2011). This led us to think that psychosocial research may make sense in a sport that contains normative foundations unlike those in most team sports. For example, it is a spirit of self-control that enables UF players to develop ethical perspectives related to themselves and others on the field of play (Crocket, 2015).

Theoretical frameworks tend to be biased because they focus on understanding cognitions when other psychological constructs may also be important in team sports. Self-refereeing creates the responsibility to play sport by the rules for sporting truth, the pleasure of play. Although more research is needed k to understand its implications, we believe that insights related to self-refereeing should be applied in different contexts. Goal orientations or motivation and achievement orientations are identified in most of the articles (Duda and Nicholls, 1992). One strategy that can help teachers, students, coaches, and sports lovers make successful behavior change is to apply this sports characteristic.

Regarding clarity of definitions, there is a lack of precision over some operational terms and classification of playing levels between studies. For example, the classification of 'novice' players ranged from novice UF players (Latinjak et al., 2018) to university UF players (Smith et al., 2013). In this sense, more clarity is necessary for defining player levels in the literature (Swann et al., 2015). It is also important for coaches to know about, understand, and enhance the characteristics of UF (self-regulation, self-refereeing, autonomy) as a key factor that make it possible to interpret collective games in ways that enhance self-regulation, self-refereeing, and autonomy so that participation leads to more “self-determined” behaviors. Therefore, it is essential to know the motivational determinants for the practice of UF. Finally, the use of cross-sectional research designs typically generates biased estimations of longitudinal mediation parameters even when samples are large. Additionally, cross-sectional designs in quantitative literature limit our ability to establish causal relationships between psychosocial factors and performance (Maxwell et al., 2011).

In terms of practical applications, as outlined at the start of this discussion, we have identified important UF characteristics that should be highlighted. First, there may be value in checking certain psychological characteristics among UF players such as friendship (Méndez-Giménez et al., 2012). With interesting rules and the atmosphere of friendship and respect that accompanies it, UF attracts players of all nationalities, ages, and both sexes (Piepiora et al., 2020). Second, interventions for increasing the sports education achievement goals and social goals to fulfill students' basic psychological needs and to promote fair play can take many forms and can be undertaken in various settings. This is helpful when interventions target individuals, small groups, and teams as we found that communication is a mediator in teamwork (Smith et al., 2013; Bosselut et al., 2018).

The present study has added original information to the current body of literature by highlighting trends in the field of sport psychology applied to UF, and by providing an exhaustive methodological appraisal of the included studies. This can assist researchers in conducting future studies on these dimensions or other psychological correlates.

### Limitations

Despite some limitations, this SR study helps to understand the lines of investigation that have been used to study UF and how to improve understanding of the game and its inherent psychological behaviors. Due to the limited number of articles related to UF, there are opportunities to gain an additional understanding of the related psychological aspects. Therefore, it is important to establish a global view around this specific area of study related to a self-referred sport. Due to the characteristic of the modality and the studies carried out so far, these dimensions seem to be attractive and should be explored in future studies, considering the introduction of other psychological related dimensions (i.e., task cohesion, group cohesion, leadership, teamwork, sportsmanship, goal orientation) as they are related to gender identities, player interaction, sports landscape, and peace culture.

### Practical Applications

Furthermore, the identification and analysis of UF characteristics and environment can be used to enhance players/students and team's performance. The results revealed that group goals and promoting teamwork significantly predicted social cohesion and that teamwork and task cohesion was mediated by communication. UF is characterized by communication between all players, whether they are from the same team or the opposing team. In summary, the current study revealed real-time information about the game and its rules. This is important because UF is one of the few team sports worldwide that are self-referred by participants.

## Conclusion

The current study provided a systematic review of the psychological domains associated with UF. We identified lines of investigation, but none takes a specific approach to self-refereeing and the use of the SOTG game sheet. Finally, we found that group goals and promoting teamwork significantly predicted social cohesion and that teamwork and task cohesion was mediated by communication. In summary, the current study provides real-time knowledge about the game and its rules as they exist in one of the few team sports that is self-refereed. There seems to be a differentiation in the players' awareness of the game using the *Spirit of the Game* sheet as the main differentiating factor. Therefore, there is a need to clarify the motivational self-talk and instructional produced better performance than a control condition for a strength task. An exciting avenue for future research would be important to examine and compare the *Spirit of the Game* with psychological correlates.

## Data Availability Statement

The raw data supporting the conclusions of this article will be made available by the authors, without undue reservation.

## Author Contributions

JA, RR-G, RA, JC, PT, JV-d-S, and GF: conceptualization, investigation, and resources. JA, RR-G, JV-d-S, and GF: data curation and formal analysis. JA and JV-d-S: funding acquisition. JA, RR-G, RA, JV-d-S, and GF: methodology. JA, JV-d-S, and GF: project administration. All authors contributed to the article and approved the submitted version.

## Conflict of Interest

The authors declare that the research was conducted in the absence of any commercial or financial relationships that could be construed as a potential conflict of interest.

## Publisher's Note

All claims expressed in this article are solely those of the authors and do not necessarily represent those of their affiliated organizations, or those of the publisher, the editors and the reviewers. Any product that may be evaluated in this article, or claim that may be made by its manufacturer, is not guaranteed or endorsed by the publisher.
